# The Role of ^18^F-FDG PET/CT in Detecting Ovarian Cancer Recurrence in Patients with Elevated CA-125 Levels

**DOI:** 10.4274/mirt.galenos.2018.00710

**Published:** 2019-03-19

**Authors:** Arzu Cengiz, Zehra Pınar Koç, Pelin Özcan Kara, Yakup Yürekli

**Affiliations:** 1 Aydın Adnan Menderes University Faculty of Medicine, Department of Nuclear Medicine, Aydın, Turkey; 2 Mersin University Faculty of Medicine, Department of Nuclear Medicine, Mersin, Turkey

**Keywords:** Ovarian cancer, 18F-FDG PET/CT, tumor, markers

## Abstract

**Objectives:**

To investigate the role of ^18^F-FDG positron emission tomography/computed tomography (PET/CT) in detection of recurrence in ovarian cancer patients with increased CA-125 levels.

**Methods:**

Fifty-two patients (30-80 years old, mean: 58.5±10.6 years) who had been histopathologically diagnosed with ovarian cancer, underwent ^18^F-FDG PET/CT imaging for re-staging due to elevation of CA-125 levels were included in this retrospective study. ^18^F-FDG PET/CT findings were compared with histopathological, radiological and clinical follow-up results.

**Results:**

CA-125 levels ranged between 35.2-2740 U/mL (N: 0-35 U/mL). Recurrent disease was detected in 45 of 52 patients on PET/CT imaging. There were three false negative and one false positive result. In addition to abdominal and pelvic lesions, 14 distant metastatic lesions (brain, lung, liver and bone metastasis) were identified correctly on PET/CT imaging. Sensitivity, specificity, positive and negative predictive value and accuracy of ^18^F-FDG PET/CT were calculated as 94%, 75%, 98%, 50% and 96%, respectively.

**Conclusion:**

^18^F-FDG PET/CT is a useful imaging method that can be used in detection of ovarian cancer recurrence in patients with elevated CA-125 levels. Since this modality offers whole body imaging, distant metastases could be detected in addition to abdominal and pelvic lesions thus contributing to patient management.

## Abstract (tr)

**Amaç:**

Bu çalışmanın amacı, serum CA-125 düzeylerinde artış olan over kanserli hastalarda rekürrens saptamada ^18^F-FDG pozitron emisyon tomografi/bilgisayarlı tomografinin (PET/BT) rolünü araştırmaktır.

**Yöntem:**

Bu retrospektif çalışmaya histopatolojik olarak over kanseri tanısı almış, CA-125 düzeylerinde artış nedeniyle yeniden evreleme amacıyla ^18^F-FDG PET/BT yapılan 52 hasta (30-80 yaş, ortalama: 58,5±10,6 yaş) dahil edildi. ^18^F-FDG PET/BT bulguları histopatolojik bulgular veya radyolojik ve klinik izlem sonuçlarıyla karşılaştırıldı.

**Bulgular:**

CA-125 düzeyleri 35,2-2740 U/mL (N: 0-35 U/mL) aralığındaydı. PET/BT görüntülerinde rekürren hastalık 52 hastanın 45’inde gösterildi. Üç yanlış negatif, bir yanlış pozitif sonuç elde edildi. Abdominal ve pelvik lezyonlara ilave olarak 14 uzak metastaz (beyin, akciğer, karaciğer ve kemik metastazı) PET/BT ile doğru olarak gösterildi. Duyarlılık, özgüllük, pozitif ve negatif öngörü değeri ve doğruluk sırasıyla %94, %75, %98, %50 ve %96 idi.

**Sonuç:**

^18^F-FDG PET/BT, serum CA-125 düzeylerinde artış olan over kanserli hastalarda rekürrens saptamada kullanılabilecek yararlı bir yöntemdir. Tüm vücut görüntüleme yöntemi olması nedeniyle abdominal ve pelvik lezyonların yanında uzak metastazları da saptayarak bu hastaların izlemine katkıda bulunabilir.

## Introduction

Ovarian cancer is the fourth leading cause of cancer death among women (1). It is usually diagnosed at advanced stages thus having poor prognosis. In spite of effective treatment and complete response, recurrence may occur in 50-80% of these patients (2,3,4). Early detection of recurrence is important for patient management.

CA-125 is a high-molecular weight glycoprotein that is expressed at the cell-surface of epithelial cells. Serum CA-125 levels are the reference method for the detection of ovarian carcinoma recurrences with a very high positive predictive value (PPV). Nevertheless, CA-125 is not specific for ovarian cancer in addition to not being sensitive especially for small-volume disease (5,6). 

^18^F-FDG positron emission tomography/computed tomography (PET/CT) is a noninvasive, highly accurate imaging method both in staging and in follow-up of many cancers including ovarian cancer. ^18^F-FDG PET/CT has a very high sensitivity rate (85-100%) for detection of recurrence in ovarian cancer (7). 

The aim of this retrospective study is to investigate the role of ^18^F-FDG PET/CT in detection of recurrence in ovarian cancer patients with increased CA-125 levels.

## Materials and Methods

### Patient Population

All patients who underwent ^18^F-FDG PET/CT for restaging due to high CA-125 levels (N: 0-35 U/mL) from March 2013 to December 2016 were retrospectively evaluated. A total of 52 patients (30-80 years; mean 58.5±10.6) were analyzed in two different institutions. All patients had undergone surgery (3-94 month ago) and chemotherapy or radiotherapy prior to PET/CT imaging. ^18^F-FDG PET/CT findings were compared with histopathological, radiological and clinical follow-up findings in at least 6 months.

The Local Ethics Committee of Adnan Menderes University approved the study (protocol number: 2018/1487, date: 27.09.2018).

### ^18^F-FDG PET/CT Imaging

All patients’ fasting blood sugar levels were less than 180 mg/dL prior to imaging. After intravenous administration of 270-370 MBq (7.3-10 mCi) ^18^F-FDG, patients rested in a quiet room. Oral contrast was given to all patients. ^18^F-FDG PET/CT imaging was performed after a resting period of 60 minutes by using Siemens (Biograph mCT 20) and General Electric (GE, Discovery 610) PET/CT scanners. The CT scan data were collected at 120 kV and 50 mAs. The PET acquisitions were obtained from the head to the midthighs at the rate of 2 minute per frame. 

All ^18^F-FDG PET/CT imaging were evaluated visually and semi-quantitatively by two nuclear medicine physicians. For semi-quantitative evaluation, maximum standardized uptake values (SUV_max_) were calculated for all pathological lesions. The lesions with a SUV_max_≥2.5 at the site of pathologic changes on CT imaging were accepted as malignant lesions.

### Data Analysis

PET/CT findings were compared with histopathologic findings (n=10) and serial conventional imaging methods and/or clinical follow-up results (n=42). If the lesion could not be histopathologically confirmed then those with decreased CA-125 levels following ovarian cancer treatment (chemotherapy or radiation therapy) and/or lesions verified by serial imaging methods including PET/CT were accepted as true positive (TP). If PET/CT findings were normal and no recurrence was detected during serial imaging or clinical follow-up then the result was classified as true negative (TN). If PET/CT findings were normal but recurrence was detected by serial imaging methods or clinical follow-up, then the results were defined as false negative (FN). Positive PET/CT results that were proved to be benign or due to a secondary malignancy were classified as false positive (FP). Patients who had both TP and FP findings were classified as TP in the patient based analysis.

### Statistical Analysis

The sensitivity, specificity, PPV and negative predictive values (NPV) and accuracy were calculated by standard statistical formulas.

## Results

A total of 52 patients with a diagnosis of ovarian cancer were included in the study. The main tumor type was serous carcinoma/adenocarcinoma (n=39, 75%), followed by clear cell carcinoma (n=3, 6%), endometroid carcinoma (n=3, 6%), mucinous carcinoma (n=3, 6%), undifferentiated carcinoma (n=2, 4%), granulosa cell tumor (n=1, 2%) and primitive neuroectodermal tumor (n=1, 2%). CA-125 levels ranged between 35.2-2740 U/mL (mean 341±564 U/mL).

^18^F-FDG PET/CT detected a hypermetabolic nodular lesion in the lung suggesting metastasis in one patient. Serial contrast-enhanced CT scans did not reveal any nodule following non-specific treatment and CA-125 levels also decreased, therefore, the PET/CT result was accepted as FP.

There were 3 FN results in the study: In one patient there was a hypometabolic cystic lesion on pelvic images but CA-125 levels decreased after chemotherapy (patient no: 8). In another patient PET/CT imaging did not show any lesions except mildly hypermetabolic (SUV_max_: 2.7) millimetric lymph nodes with benign appearance in the mediastinum suggesting reactive enlargement, however, serial PET/CT imaging detected progression and CA-125 levels increased progressively (patient no: 17). In the third patient, PET/CT imaging did not reveal any hypermetabolic lesions but serial CT imaging detected local recurrence (patient no: 40). In this patient, recurrence was confirmed by biopsy during follow-up.

**Figure 1:**
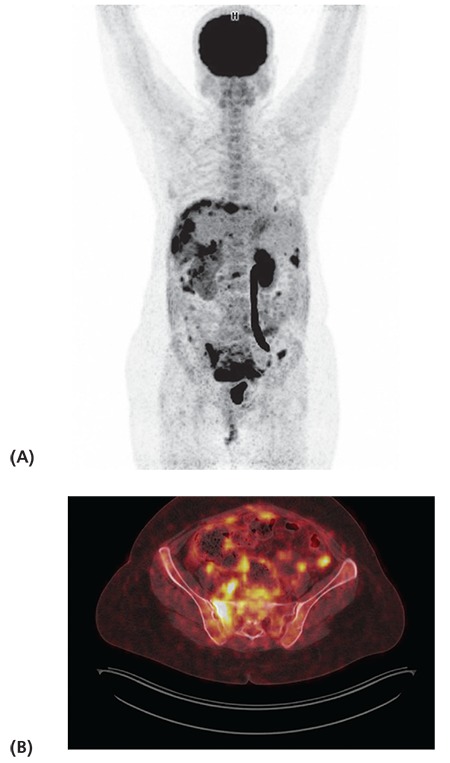
Maximum intensity projection (A) and axial fused positron emission tomography/computed tomography (B) images of a 47-year-old patient with stage 1B serous ovarian carcinoma (patient no: 24) show widespread peritoneal involvement and mesenteric implants (SUV_max_: 15.5), lymph nodes (SUV_max_: 7.4), hypermetabolic lytic lesions in the sacrum and L3 vertebra (SUV_max_: 16.5) suggestive of metastasis. The patient received chemotherapy, her serial positron emission tomography/ computed tomography images showed regression and CA-125 levels decreased progressively

**Figure 2:**
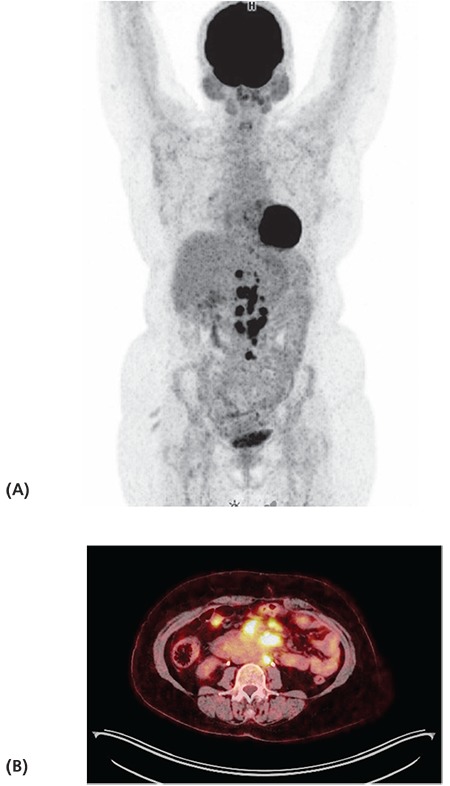
Maximum intensity projection (A) and axial fused positron emission tomography/computed tomography (B) images of a 49 yearold patient with serous carcinoma (patient no: 26) show increased ^18^F-FDG uptake in para-aortic and celiac lymph nodes (SUV_max_: 18.3) and mesenteric implants (4.2). Peritoneal biopsy confirmed malignancy in this patient

Fourteen distant metastasis were detected correctly in 12 patients on ^18^F-FDG PET/CT imaging (8 of them liver, 2 bone, 2 lung, one pleura, and one brain metastasis). Two patients with positive ^18^F-FDG PET/CT findings are illustrated in Figures 1, 2. 

According to patient-based analysis; the sensitivity, specificity, PPV, NPV and accuracy of ^18^F-FDG PET/CT in detecting ovarian cancer recurrence in patients with elevated CA-125 levels were calculated as 94%, 75%, 98%, 50% and 96%, respectively. 

Detailed results of PET/CT imaging and final diagnosis of all patients are shown in Table 1.

The patients were divided into two different groups as those with CA-125 elevation less than 100 U/mL (n=22) and those with ≥100 U/mL (n=30). The sensitivity and specificity rates of PET/CT imaging according to CA-125 levels are shown in Table 2. Because there is no TN result in patients with CA-125 levels ≥100, specificity could not be calculated in this group.

## Discussion

Early detection of tumor recurrence is important in ovarian cancer patients due to its close relation with prognosis and the choice of appropriate treatment. Even after effective treatment and complete response, the recurrence rate is 50-80% in these patients (2,3,4,8). 

In addition to clinical examination and imaging modalities, CA-125 measurements are also used for monitoring disease progression in patients with ovarian cancer. Nevertheless, several benign diseases such as infections may cause elevation in CA-125, and it is not reliable in detecting disease recurrence due to its high FN results (6,9). In this study, the patient with a TN finding had an infection at the operation site and the high CA-125 level was attributed to this infection.

Although, CT and magnetic resonance imaging (MRI) are the most commonly used imaging methods to detect recurrent ovarian cancer; their contribution is limited in small-volume recurrent lesions or metastatic deposits on visceral surfaces. CT has low sensitivity (25-50%) for detection of peritoneal metastases smaller than 1 cm (7,10). 

^18^F-FDG PET/CT has been shown to be superior to CT and MRI in detection of recurrent ovarian cancer. It might specify recurrent ovarian cancer approximately 6 months prior to CT (11). In a meta-analysis, the authors evaluated diagnostic performance of CA-125, PET, PET/CT and MRI in 34 recurrent ovarian cancers, and they reported that CA-125 had the highest specificity (93%) while PET/CT had the highest sensitivity (91%). They also showed that diffusion weight MRI is showing promise in detecting small volume peritoneal disease and may be used complementary to PET. The pooled sensitivity and specificity did not show any statistical significance between PET alone and PET/CT in this study (12). 

The reported sensitivity and specificity of ^18^F-FDG PET/CT imaging ranged from 80-100% and 42%-100%, respectively, in detecting recurrent disease (4,7,13,14). Fagotti et al. (15) reported the sensitivity, specificity, NPV, PPV, and accuracy of ^18^F-FDG PET/CT in recurrent ovarian cancer as 93.0%, 55.6%, 83.3%, 76.9% and 78.6%, respectively. In the same study, authors reported the sensitivity, specificity, PPV, NPV and accuracy rates for laparoscopy as 95%, 64%, 80.8%, 88.9% and 83.1%, respectively (15). In another study, Sari et al. (16) investigated the role of ^18^F-FDG PET/CT in recurrent ovarian cancer with high tumor markers or suspicious lesions on CT and they reported the sensitivity, specificity and accuracy of PET/CT as 96.1%, 100% and 97%, respectively. 

In this study, sensitivity, specificity, PPV, NPV and accuracy of ^18^F-FDG PET/CT in detecting ovarian cancer recurrence in patients with elevated CA-125 levels were 94%, 75%, 98%, 50% and 96%, respectively, which were concordant with the literature. Compared to previous studies, NPV is relatively low in our study. Cystic or necrotic lesions and low grade tumor may result in FN ^18^F-FDG PET/CT imaging findings (4). A hypometabolic cystic lesion on pelvic images was one of the FN results. ^18^F-FDG PET has a lower sensitivity in detection of primary or recurrent mucinous carcinoma, but all FN results were from patients with a diagnosis of serous carcinoma in this study. These results may be attributed to low grade tumor or early disease progression and small lesion size at the time of PET/CT imaging. In accordance with our results, Risum et al. (17) found high sensitivity (97%) for ^18^F-FDG PET/CT in patients with high CA-125 levels although they reported relatively low NPV rate (43%) due to micro or cystic/mucinous lesions. 

Recurrences were primarily detected in peritoneal cavity and retroperitoneal lymph nodes in 75% of patients with ovarian cancer (18). In our study, we concordantly detected peritoneal and retroperitoneal metastases in majority of patients (41/52, 79%). PET/CT may not be able to demonstrate diffuse peritoneal involvement or small volume disease and small or necrotic lymph nodes (19,20). Rubini et al. (21) investigated the role of ^18^F-FDG PET/CT in diagnosis of peritoneal carcinomatosis in patients with ovarian cancer and they reported the sensitivity, specificity, accuracy, PPV and NPV of ^18^F-FDG PET/CT as 85%, 92.31%, 88.61%, 91.89% and 85.71%, respectively. In a meta-analysis which included eighteen studies, authors compared the diagnostic performances of CT, MRI and PET/CT for detection of metastatic lymph nodes in patients with ovarian cancer and they concluded that ^18^F-FDG PET/CT is more accurate (sensitivity, 73.2%; specificity, 96.7%) than CT and MRI (sensitivity, 42.6% and 54.7%; specificity, 95.0% and 88.3%) (22).

One of the main advantages of PET/CT is the information about the extent and location of recurrence. Early diagnosis of recurrence and exact localization of metastatic disease are crucial for determination of the best treatment strategy. In a study, the authors reported that PET/CT findings changed clinical management in 58% of patients (23). We detected fourteen distant metastasis correctly in 12 patients with ^18^F-FDG PET/CT in addition to abdominal and pelvic peritoneal metastasis in our study.

### Study Limitations

The main limitation of our study is its retrospective design. Patients were included from two different institutions and imaging techniques could not be standardized. Besides, pathological confirmation of ^18^F-FDG positive lesions could not be performed in all patients.

## Conclusion

In conclusion, ^18^F-FDG PET/CT is a useful imaging method that can be used in detection of ovarian cancer recurrence in patients with elevated CA-125 levels. Since this modality offers whole body imaging, distant metastases could be detected in addition to abdominal and pelvic lesions thus contributing to patient management.

## Figures and Tables

**Table 1:**
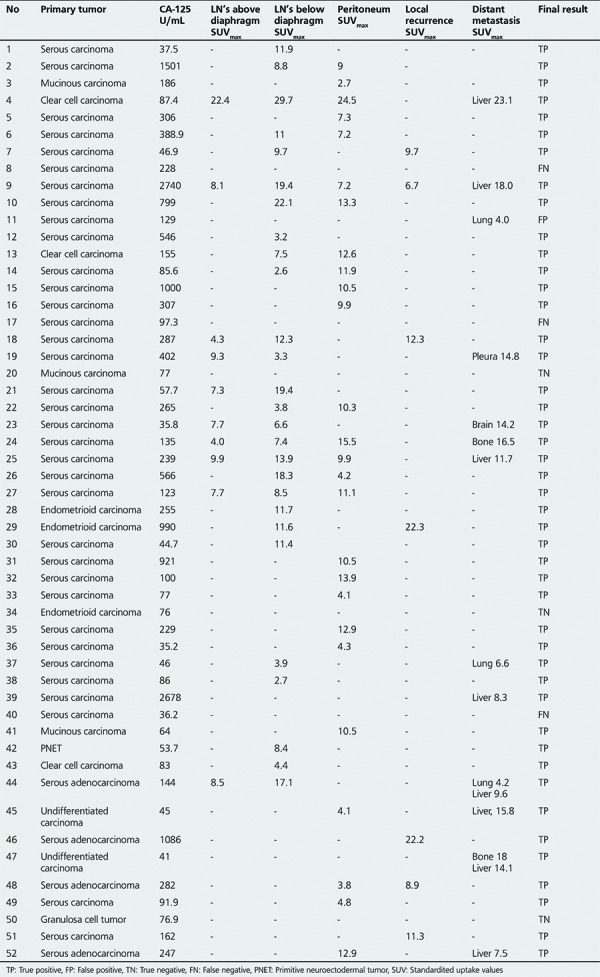
Positron emission tomography/computed tomography imaging findings and final diagnosis of all patients

**Table 2:**
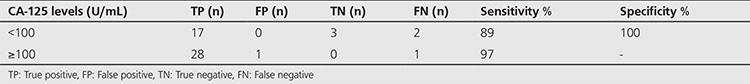
Detailed results of ^18^F-FDG positron emission tomography/computed tomography according to CA-125 levels
